# Effect of Short-Term Mobile Phone Base Station Exposure on Cognitive Performance, Body Temperature, Heart Rate and Blood Pressure of Malaysians

**DOI:** 10.1038/srep13206

**Published:** 2015-08-19

**Authors:** F. Malek, K. A. Rani, H. A. Rahim, M. H. Omar

**Affiliations:** 1 School of Electrical System Engineering, Universiti Malaysia Perlis (UniMAP), Pauh Putra, Arau, Perlis 02600, Malaysia; 2 School of Computer and Communication Engineering, Universiti Malaysia Perlis (UniMAP), Pauh Putra, Arau, Perlis 02600, Malaysia; 3 Faculty of Engineering Technology, Universiti Malaysia Perlis (UniMAP), Pauh Putra, Arau, Perlis 02600, Malaysia

## Abstract

Individuals who report their sensitivity to electromagnetic fields often undergo cognitive impairments that they believe are due to the exposure of mobile phone technology. The aim of this study is to clarify whether short-term exposure at 1 V/m to the typical Global System for Mobile Communication and Universal Mobile Telecommunications System (UMTS) affects cognitive performance and physiological parameters (body temperature, blood pressure and heart rate). This study applies counterbalanced randomizing single blind tests to determine if sensitive individuals experience more negative health effects when they are exposed to base station signals compared with sham (control) individuals. The sample size is 200 subjects with 50.0% Idiopathic Environmental Intolerance attributed to electromagnetic fields (IEI-EMF) also known as sensitive and 50.0% (non-IEI-EMF). The computer-administered Cambridge Neuropsychological Test Automated Battery (CANTAB *eclipse*^TM^) is used to examine cognitive performance. Four tests are chosen to evaluate Cognitive performance in CANTAB: Reaction Time (RTI), Rapid Visual Processing (RVP), Paired Associates Learning (PAL) and Spatial Span (SSP). Paired sample t-test on the other hand, is used to examine the physiological parameters. Generally, in both groups, there is no statistical significant difference between the exposure and sham exposure towards cognitive performance and physiological effects (*P’s* *>* *0.05)*.

The existence of radio frequency electromagnetic field (RF EMF) exposure effects from the mobile phone base station and their implications have become the subject of ongoing researches and have not been established sufficiently to be absorbed as a standard criterion. Most of the studies reveal that UMTS, Third-Generation (3G) technology focuses on the effects of human cognitive functions. Individuals who report that they are sensitive to electromagnetic fields often experience cognitive impairments which they believe are due to exposure to mobile phone technology. Furthermore, they also complaint on headache as they perceive such symptom is caused by the RF EMF exposure^1^.Headache is an important warning sign that body temperature is rising to a risky levels, suggesting that when RF heats biological tissues, body temperature and other vital physiological parameters such as heart rate and blood pressure may change^2^. Therefore, there is an urgent need to study the issue among Malaysians as to shed some light and make them aware of mobile technology with regards to mobile base stations and their safety when living near to the base stations. Thus, this paper aims to determine whether the relation between electromagnetic fields (EMF) and the subjective complaints together with physiological changes and cognitive performance are associated with electronic exposure.

## Methodology

### Subjects

Participants are recruited through local advertising, action groups, and words of mouth. They are required to report to the research committee for an interview, attend and practice the cognitive tests prior to the experiment. Number of participants is determined by using G*Power analysis^3^. In G*Power, a total sample size of 195 is required to detect significant of the model at α = 0.05 with 0.26 small effect. The sample size is enlarged to 200 samples to get better accuracy:100 IEI-EMF and 100 non-IEI-EMF categories. The subjects for this study are classified into two groups. Sensitive group denotes the group of subjects that have previously reported complaints and have attributed these complaints to GSM or UMTS exposure (IEI-EMF) while non-sensitive group denotes the reference group, namely a group of subjects without any complaints (non-IEI-EMF category). The experiment excludes shift workers within the last month before the experiment is conducted. On their first appointment, all subjects have to fill out a questionnaire to verify the exclusion and matching criteria (age in decades, sex, and residential area). Written consent is obtained from all subjects. The study is approved by Ethical Committee, Universiti Malaysia Perlis, Malaysia. The methods are carried out in accordance with the approved guidelines.

### Experimental Setup

This study focuses on controlled power received by each subject to 1 V/m, both from GSM and UMTS field exposure (based on actual approximated exposure measurement readings in Malaysia). The signal radiation setup used in the study is similar to the signals used by other studies^4^,^5^,^6^,^7^. The experiment is performed in an RF shielded room, lined using microwave absorbing sheets in the Electromagnetic HyperSensitivity (EHS) Laboratory located in Politeknik Syed Sirajuddin, Perlis, Malaysia. The room is furnished with a wooden table, a flat-screen monitor, a chair and sofa, as well as the GSM and UMTS base station antenna. In order to determine shielding effectiveness within the room, the free space measurement technique (based on Standard No. IEEE 299(1)) using two horn antennas is applied. The shielding effectiveness of RF-shielded room is about 48 dB at the tested frequency range. Both of the experimenters and the subjects are present in the shielded room.

The base station antenna (Kathrein 800 10046/GSM900/GMS1800/UMTS) is placed at 1.5 m from the ground floor and 2 m distance from the subjects. Four exposure signals are tested which include GSM900, GSM1800, UMTS, and Sham. The GSM and UMTS used in this study are similar to the signals used in other studies^4^,^7^,^8^. The GSM900 has a frequency of 945 MHz with a power flux density of 280 W/m^2^ while the GSM1800 contains a frequency of 1840 MHz with a power flux density of 250 W/m^2^ over the area in which the subject is positioned. The GSM signal format consists of eight time slots; each feature consists a duration of 546.5 *μ*sec, leading to a total frame duration of 4.372 msec. The guard time between the time slots of 30.5 *μ*sec is inserted into each GSM frame. To simulate the changes in the power level of channels, time slots of 0, 1, 2, and 3 are set to ‘on’ (transmitted) whereas the time slots of 4, 5, 6, and 7 are chosen to be ‘off’. This results in a nearly pulsed modulated field with a frequency of approximately 217 Hz. The power level drop is about 50 dB between the active state (the burst) and the inactive state (the guard).

The UMTS signal has a frequency of 2140 MHz with a chip rate 3.84 microchips/sec and a power flux density of 380 W/m^2^ measured over the area where the subject is seated. The UMTS signal comprises of four controls and synchronization channels which are primary synchronization channels of −8.3 dB below total RF power, secondary synchronization channel of 8.3 dB, primary common control physical channel of −5.3 dB, and common pilot channel of −3.3 dB. Under Sham exposure condition, no signal is transmitted. The signals are generated using a Rohde and Schwarz SMBV100A, connected to the GSM and UMTS base station antenna through the coaxial cable. The exposure is consistently monitored in ensuring the stability of the exposure system. The background RF radiation levels (80 MHz–4 GHz) are measured before and after the measurement and the levels are well below 1 mV/m over the exposure area.

Physiological changes of subjects are monitored before and after the exposure session. An exposure session is designed under counterbalanced randomized single-blind conditions which will be in a randomized crossover design. During the single-blind tests, the participants will not be notified of which exposure is being generated. Before exposure, subjects are given a briefing and training in an office room and will then be escorted to the exposure chamber. Subjects are exposed with four different types of RF exposure which are GSM900, GSM1800, UMTS, and sham (no exposure). For each type of exposure, the experiment is divided into three sessions which involve pre-exposure, during exposure, and post exposure.

A complete schedule has been generated manually to ensure the orders are balanced for all participants. To make sure the data is randomly allocated, a set of random number (1 to 200) has been generated. Participants are not informed about the order of the exposure that has been scheduled for them. The subjects receive every treatment (Sham, GSM900, GSM1800 and GSM2100). It carries an important assumption in which the effect of treatment received in each period is not affected by treatment received in the previous period.

Two outcome measures are chosen in measuring Paired Associates Learning (PAL). PAL Total errors (adjusted) measure the total number of errors across all assessed problems in all stages with an adjustment of each stage not attempted due to previous failure while PAL Mean errors (to succeed) measure mean number of errors made before the stage was successfully completed. Reaction Time (RTI) measures the participant’s speed of response towards a visual target. The participants have to hold the pressure pad down, then release it and touch the screen as quickly as possible when a yellow dot appears at the centre. Two outcome measures are chosen in measuring Reaction Time (RTI) which are Five-Choice movement time and Five-Choice reaction time. Reaction Time (Five-choice reaction time) is the speed with which the subject releases the press pad in response to the onset of a stimulus in a single location, whereas Five-Choice movement time is the time acquired to touch the stimulus on the screen after the press pad is released. Both outcomes are determined in milliseconds. The fastest time it takes, the better outcome it will be.

Rapid Visual Processing measures general performance of vigilance and attention. RVP A’ is used as the outcome to measure the performance. RVP A’ is the signal detection measure of sensitivity to the target regardless of response tendency (range 0.00–1.00; bad to good). The higher the range that the subject gets, the better it will be. Clinical mode of Spatial Span length has a possible of 9 maximum score. Spatial Span length is the longest sequence successfully recalled by the subject and the subject has three attempts at each level.

Continuous variables are summarized by using descriptive statistics, which include mean and standard error. Two-way analysis of variance is used to detect differences in cognitive performance across exposure. In order to measure whether the mean value from each physiological parameter varies over two conditions (pre- and post-condition), paired sample t-test is used. Next, Independent sample t-test is used to answer whether IEI-EMF and non-IEI-EMF groups differ in terms of their physiological parameters (body temperature, blood pressure and heart rate). All statistical analysis is conducted using SPSS (Statistical Package for Social Science version 11.0).

## Results

The results of the cognitive test for IEI-EMF (Sensitive) group and non-IEI-EMF (Normal) group can be seen in Table 1. The primary analysis shows a significant (*P* < *0.05*) effect of learning for PAL Total errors (adjusted) and PAL mean errors (to succeed) with no significant difference between signals (Sham, GSM900, GSM1900 and UMTS) *(P* > *0.05)* and no significant interaction between group and signal (*P* > *0.05*) but a significant difference in group *(P* < *0.05).* Thus, PAL performances (Cognitive) of both groups (Sensitive and Normal) are not affected by Signal even though there is a significant difference in PAL performance between groups. The results of the RTI Five choice test (movement time and reaction time) shows a significant effect *(P* < *0.05)* with no significant difference between Normal and Sensitive *(0050* > *0.05)* among all four signals *(P* > *0.05)* as well as no significant interaction between groups and types of signals *(P* > *0.05).* Thus, there is no significant difference on RTI performance when subjects are exposed to the Signal. Normal Subjects in UMTS signal has an average of slower reaction time than other signals that indicate “good performance on RTI”.

On average, the overall mean of RVP A’ in range is between 0.897 and 0.932 which is closed to 1.00. It reveals a good performance of RVP. The signal detection measure of sensitivity to the target that slightly increases from Sham to UMTS can be seen in Table 1. The results in Table 1 demonstrates that there is no significant difference between Normal and Sensitive groups *(P* > *0.05),* no significant difference among all four signals *(P* > *0.05)* and no significant interaction between groups and signals *(p* > *0.05)* on Span length. Thus, the test of cognitive performance on RVP is not affected by any Signal. In addition, both groups reach the maximum score of 9 with a mean length *(M* = *7. 00).*

The physiological data is subjected to two sets of analysis. The purpose of the first analysis is to determine whether there is a significant difference between the average values of the body temperature, heart rate and blood pressure under two different conditions. The test is based on the paired differences between these two values (pre-exposure and post-exposure). The second analysis is to compare whether Normal and Sensitive subjects have different average body temperature, heart rate and blood pressure. Mean and standard error for body temperature (BT), heart rate (HR), systolic blood pressure (BPS) and diastolic blood pressure (BPD) are computed for the Normal and Sensitive subjects. Table 2 is shown below to illustrate the mean difference of two conditions (pre- and post-exposure), standard errors, and *p-values.* Across all 200 subjects, mean of heart rate is between 74–83 beats per minute in which heart rate of subjects are classified as in normal resting heart rate (60–100 beats per minutes). The paired sample t-test (pre-exposure and post-exposure) that is applied to investigate the effects of short term GSM and UMTS for body temperature (BT) and blood pressure is not significant (*P* *>* *0.05)* which indicates that body temperature and blood pressure are not affected by short term GSM and UMTS. The results of the paired sample t-test can be seen in Table 3. On the other hand, for the heart rate (HR), there is a statistically significant difference between the pre- and post-exposure sessions, *p* *<* *0.05.* The heart rate of subjects clearly decreases over the course of the study; on average, about 3 beats per minute. During both conditions (pre-exposure and post-exposure) of each Signal including Sham, *P’s* *>* *0.05,* there is no significance between group differences which indicate that in average, the physiological parameter of Normal and Sensitive subjects remain unchanged or constant after they are exposed to all signals.

## Conclusion

This study examines the existence relation between electromagnetic fields (EMFs) and the subjective complaints together with physiological changes and cognitive performance associated with an electromagnetic stimulus. In conclusion, the researchers report that they discover no significant effects of short-term GSM and UMTS base station signal exposure on cognitive performance, body temperature, blood pressure and heart rate of Malaysians. In both the IEI-EMF and non-IEI-EMF groups, there are no significant differences of the mean value in body temperature, blood pressure and heart rate between sham real exposures to an electromagnetic stimulus before and after exposure. These results are paralleled with the previous studies using a similar experimental design to investigate the effects of radiation on the physiological factor as they found out that the radiation did not affect the heart rate of the subjects^8^,^9^. Control individuals exhibit significantly lower levels of heart rate than the IEI-EMF individuals under both GSM900 and GSM1800 signals between before and after exposures. However, the heart rate does not differ between the two groups in GSM900 and GSM1800 signals regardless of exposure conditions. Moreover, under UMTS exposure, both IEI-EMF and non-IEI-EMF groups show a reduction in heart rate level from before to after exposure, but no difference is observed between the two groups either before or after exposure, which is consistent with recent finding^9^. This concludes that the short-term base station signals of GSM and UMTS do not affect the heart rate of either normal or sensitive subjects, indicating that no negative health effects can be associated with EMF exposure.

## Additional Information

**How to cite this article**: Malek, F. *et al.* Effects of Short-Term Mobile Phone Base Station Exposure on Cognitive Performance, Body temperature, Heart Rate and Blood Pressure of Malaysians. *Sci. Rep.* **5**, 13206; doi: 10.1038/srep13206 (2015).

## Figures and Tables

**Table 1:** The Results for Cognitive Performance.

	**Sham**		**GSM900**		**GSM1800**		**UMTS**		**p-value**
	**Normal Mean (S.E)**	**Sensitive Mean (S.E)**	**Normal Mean (S.E)**	**Sensitive Mean (S.E)**	**Normal Mean (S.E)**	**Sensitive Mean (S.E)**	**Normal Mean (S.E)**	**Sensitive Mean (S.E)**	(Group),(Signal) (Group*Signal)
**PAL Total errors (adjusted)**	7.94 (0.89)	7.50 (0.74)	5.14 (0.56)	6.06 (0.78)	5.09 (0.58)	4.68 (0.51)	5.34 (0.55)	6.20 (1.55)	(0.01*),(0.69), (0.74)
**PAL Mean errors** (**to succeed)**	1.58 (0.18)	1.49 (0.15)	1.03 (0.11)	1.21 (0.15)	1.02 (0.12)	0.94 (0.1)	1.07 (0.11)	1.08 (0.18)	(0.01*),(0.98), (−0.76)
**RTI Five-choice movement time**	575.40 (17.99)	570.22 (15.87)	575.47 (20.32)	587.60 (16.75)	568.57 (17.10)	563.19 (17.80)	559.06 (17.86)	546.41 (12.98)	(0.392),(0.820), (0.905)
**RTI Five-choice reaction time**	346.07 (7.07)	345.74 (7.28)	351.15 (7.04)	360.15 (7.57)	348.41 (6.97)	360.32 (7.90)	343.94 (6.34)	363.41 (7.51)	(0.525),(0.050), (0.588)
**RVP A’**	0.90 (0.01)	0.92 (0.01)	0.92 (0.01)	0.92 (0.01)	0.93 (0.01)	0.93 (<0.01)	0.92 (0.01)	0.93 (0.01)	(0.00*),(0.022*), (−0.349)
**SSP Span length**	7.44 (0.15)	7.34 (0.16)	7.67 (0.14)	7.54 (0.17)	7.84 (0.15)	7.65 (0.15)	7.58 (0.16)	7.53 (0.19)	(0.164),(0.295), (0.977)

Notes: PAL: Paired Associates Learning, RTI: Reaction Time, RVP: Rapid Visual Processing, SSP: Spatial Span, S.E: Standard Error.

*p-value<0.05.

**Table 2:** The Descriptive Statistics for Physiological Parameters.

	**Sham**			**GSM900**			**GSM1800**			**UMTS**		
	**Normal Mean (S.E)**	**Sensitive Mean (S.E)**	**p-value**	**Normal Mean (S.E)**	**Sensitive Mean (S.E)**	**p-value**	**Normal Mean (S.E)**	**Sensitive Mean (S.E)**	**p-value**	**Normal Mean (S.E)**	**Sensitive Mean (S.E)**	**p-value**
**PreBT**	36.04 (0.11)	36.04 (0.06)	0.963	35.93 (0.12)	35.86 (0.12)	0.671	36.09 (0.05)	36.14 (0.23)	0.855	35.84 (0.12)	35.92 (0.16)	0.686
**PostBT**	36.08 (0.06)	36.02 (0.11)	0.633	36.58 (0.50)	36.72 (0.70)	0.874	35.86 (0.13)	36.01 (0.06)	0.296	35.88 (0.11)	36.08 (0.06)	0.115
**PreHR**	80.23 (1.35)	82.90 (1.29)	0.154	79.85 (1.35)	81.29 (1.38)	0.458	77.81 (1.26)	80.11 (1.30)	0.205	81.92 (1.65)	81.18 (1.40)	0.731
**PostHR**	80.03 (1.66)	80.51 (1.70)	0.840	75.71 (1.28)	79.17 (1.93)	0.137	74.20 (1.14)	78.06 (1.26)	0.025	77.36 (1.43)	76.80 (1.11)	0.759
**PreBPS**	127.59 (1.98)	129.18 (2.15)	0.587	127.17 (2.36)	127.07 (2.02)	0.974	126.92 (2.26)	129.22 (2.21)	0.468	124.99 (1.99)	126.25 (2.03)	0.659
**PostBPS**	126.81 (2.00)	125.44 (2.26)	0.651	125.53 (2.19)	126.36 (2.22)	0.791	125.04 (1.94)	140.01 (14.76)	0.316	124.75 (2.05)	125.05 (2.35)	0.923
**PreBPD**	80.32 (1.33)	79.36 (1.61)	0.647	78.74 (1.50)	78.15 (1.69)	0.794	80.29 (1.55)	77.92 (1.61)	0.289	79.85 (1.32)	79.08 (1.35)	0.684
**PostBPD**	87.29 (6.36)	79.10 (1.39)	0.210	86.09 (6.75)	79.29 (1.57)	0.328	79.60 (1.23)	85.87 (6.44)	0.340	80.30 (1.27)	79.60 (1.45)	0.717

**Table 3:** The Statistical Test Results for Physiological Parameters.

	**Sham**		**GSM900**		**GSM1800**		**UMTS**	
	**Normal Mean(S.E) (p-value)**	**Sensitive Mean(S.E) (p-value)**	**Normal Mean(S.E) (p-value)**	**Sensitive Mean(S.E) (p-value)**	**Normal Mean(S.E) (p-value)**	**Sensitive Mean(S.E) (p-value)**	**Normal Mean(S.E) (p-value)**	**Sensitive Mean(S.E) (p-value)**
**PreBT – PostBT**	−0.04 (0.12) (0.69)	0.02 (0.11) (0.86)	−0.65 (0.60) (0.28)	−0.85 (0.70) (0.23)	0.23 (0.13) (0.08)	0.13 (0.23) (0.58)	−0.04 (0.14) (0.77)	−0.16 (0.16) (0.32)
**PreHR –PostHR**	0.20 (1.72) (0.90)	2.39 (1.71) (0.16)	4.14 (1.39) **(<0.01)**	2.12 (1.94) (0.28)	3.67 (1.15) **(<0.01)**	2.05 (1.55) (0.19)	4.57 (1.82) **(<0.05)**	4.38 (1.42) **(<0.01)**
**PreBPS – PostBPS**	0.78 (1.65) (0.64)	3.74 (2.15) (0.08)	1.64 (1.62) (0.32)	0.71 (1.69) (0.67)	1.88 (1.84) (0.31)	−10.8 (14.2) (0.45)	0.24 (1.72) (0.89)	1.20 (2.15) (0.58)
**PreBPD – PostBPD**	−6.97 (6.48) (0.28)	0.26 (1.44) (0.86)	−7.35 (7.10) (0.30)	−1.14 (1.75) (0.52)	0.69 (1.45) (0.64)	−7.95 (6.55) (0.23)	−0.45 (1.10) (0.68)	−0.52 (1.32) (0.69)
